# Exploring a Range of Potential Factors Promoting and Constraining EF in South African Preschoolers

**DOI:** 10.1111/desc.70289

**Published:** 2026-10-14

**Authors:** Johanna Beukes, Caylee Cook, Steven J. Howard, Gaia Scerif, Christopher F. Sharpley, Robert Hart, Catherine Draper

**Affiliations:** ^1^ SAMRC/Wits Developmental Pathways for Health Research Unit, Department of Paediatrics, School of Clinical Medicine, Faculty of Health Sciences University of the Witwatersrand Johannesburg South Africa; ^2^ Early Start and School of Education University of Wollongong Wollongong NSW Australia; ^3^ Department of Experimental Psychology University of Oxford Oxford UK; ^4^ Department of Neuroscience University of New England Armidale NSW Australia; ^5^ Department of Biomedical Sciences University of New England Armidale NSW Australia

**Keywords:** executive function, preschool, socioeconomic status, South Africa

## Abstract

**Background:**

Socioeconomic disadvantage is widely associated with poorer cognitive and educational outcomes. However, emerging evidence suggests that children in low‐income contexts may demonstrate resilience in executive function (EF) development. This study examined the predictors of EF in South African preschoolers from disadvantaged communities to identify modifiable, context‐specific influences.

**Methods:**

125 Children (aged 3–5 years) and their caregivers were recruited from 15 early childhood centers in Cape Town. Children completed three tablet‐based EF tasks from the Early Years Toolbox measuring working memory, inhibition, shifting, while caregivers reported on demographic variables, household assets, child sleep, child screen time, child exposure to violence, and parenting practices. Multiple linear regressions examined predictors of child EF, with exploratory analyses on a subset (*n* = 22) for hair cortisol analysis.

**Results:**

Older child age (*β* = 0.37, *p* < 0.001), caregiver secondary education (*β* = 0.22, *p* = 0.003), and fewer sleep difficulties (*β* = –0.45, *p* = 0.042) significantly predicted higher child EF scores, together explaining 25% of variance. Household assets, screen time, violence exposure, and parenting scores were non‐significant. In the subsample, hair cortisol concentration correlated positively with EF (*ρ* = 0.55, *p* = 0.009), suggesting potential adaptive calibration under adversity.

**Conclusions:**

Findings highlight that modifiable proximal factors (e.g., caregiver education and child sleep) shape EF development more strongly than socioeconomic indicators. Results challenge the presumed universality of SES‐EF gradients and underscore the importance of culturally grounded, contextually validated measures for understanding resilience and early cognitive development in Majority World settings.

## Summary

- This study examined the predictors of executive function among South African preschoolers growing up in low‐income, high‐adversity settings.
- Findings show that caregiver secondary education and child age are significant predictors of executive function, rather than traditional socioeconomic indicators.
- Results challenge the presumed universality of the socioeconomic status–executive function gradient derived largely from high‐income contexts.
- The study underscores the need for culturally grounded, contextually validated measurement tools to better capture executive function and resilience in Majority World settings.

## 1 Introduction

Socioeconomic disadvantage has profound and lasting effects on child development, with extensive research linking lower socioeconomic status (SES) to poorer cognitive (Meng et al. 2024), educational (Falk et al. 2021, Kabasakal et al. 2020, Yang and Yang 2021), and health outcomes (Rehman et al. 2024, Iguacel et al. 2018, Marttila‐Tornio et al. 2021). Research from high‐income countries has established a link between SES and cognitive outcomes. Various studies demonstrate that children from higher‐SES backgrounds tend to have better cognitive performance, driven by factors such as enriched home learning environments, greater access to educational resources, and lower exposure to chronic stress (Last et al. 2018, Dennis et al. 2022, Takacs and Kassai 2019). Foundational abilities that support academic performance, such as executive function (EF) and self‐regulation, are found to be particularly vulnerable to adverse effects of lower SES‐related stressors, environmental instability, and reduced access to quality early childhood education (Andrews et al. 2021, Wexler et al. 2020, Qiu, and Wang, 2024).

Socioeconomic status (SES) has long been used as a catch‐all phrase to characterise resources like disposable income, housing, opportunities like employment, access to health care and education, and exposure to environmental risks such as substance abuse, violence, and criminality (Qaribilla et al. 2024, Assari and Zare 2025, Amiri and Zhao 2019, Boyce et al. 2020, Kokkinos and Myers 2021). These can alter lifetime trajectories for intellectual, behavioural, social‐emotional, and health outcomes (Acevedo‐Garcia et al. 2020). The influence of SES is so widely accepted that it has become ubiquitous as a control variable in analyses that investigate the antecedents of child outcomes. Indeed, meta‐analyses of the predictive power of SES have shown positive associations with: child cognitive abilities, academic achievement and mental well‐being (Poulain et al. 2020 Apr 14), adolescent substance use, physical health, and mental health (Walker et al. 2024), as well as substance use, health, and premature death in adulthood (Eken et al. 2024).

Yet SES is a complex construct, conflating more proximal and potentially intervention‐sensitive factors (e.g., parenting practices, caregiver education) with broader structural factors that are less readily modifiable within the family context (e.g., financial position, environmental risk, access to services) (Racine Maurice et al. 2021 Jun 21). Contemporary developmental frameworks, such as Bronfenbrenner's socioecological model, propose that children's cognitive development emerges through ongoing interactions between the child and multiple nested environmental systems, ranging from immediate caregiving environments to broader socioeconomic and cultural contexts (Ashiabi and O'Neal 2015). Within these frameworks, distal socioeconomic conditions are thought to influence developmental outcomes partly through their effects on proximal processes, including caregiving practices, daily routines, cognitive stimulation, and exposure to stress. For this reason, contemporary research has sought to identify the modifiable individual (e.g., sleep, bilingualism, engagement with music; (Drummond et al. 2012, Chaput et al. 2017), familial (e.g., maternal education, children being given meaningful responsibilities in the home, quality of home learning environment; 28–30), and contextual factors (e.g., exposure to risk, collectivist culture; (Andrews et al. 2021, Ashiabi and O'Neal 2015, Blair et al. 2011) that protect against negative trajectories and promote better life outcomes. Importantly, caregiver education may influence EF development both directly and indirectly, not necessarily through educational attainment itself, but through associated differences in literacy, cognitive stimulation, health knowledge, problem‐solving skills, and the quality of the home learning environment. In this sense, caregiver education may function both as a developmental resource and as a marker of broader social and contextual advantage. A broad range of candidate factors have been suggested, such as participation in high‐quality preschool and targeting the cognitive capacities that contribute to a broad range of later life outcomes (Smithers et al. 2018 Nov).

The promise of EF for narrowing SES‐related achievement gaps is supported by evidence that early EF contributes to a broad array of life outcomes (i.e., intellectual, behavioural, health, vocational, social), as a result of EF‐enabled resistance to impulses and distraction, holding and manipulating information in mind, and flexibly shifting thinking with situational demands (Last et al. 2018, Albert et al. 2020, Mooney et al. 2024). Furthermore, EF is malleable through everyday activities, with improvements in EF transferring to better real‐world outcomes (Isquith et al. 2014, Stamenova and Levine 2019, Howard et al. 2017). At the same time, EF have been shown to follow a similar gradient to SES, with children from higher SES backgrounds generally demonstrating stronger EF skills (Howard et al. 2017). This EF‐SES gradient has been found in both high‐income Minority World countries (i.e., economically dominant, industrialized, Westernised nations that comprise a minority of the global population), and Majority World countries (i.e., lower‐ and middle‐income regions representing the majority of the world's population) (Khan et al. 2022, Alam 2008). This implies that intervention approaches may do well to extrapolate conditions and practices from higher income contexts, to derive benefit for children in low‐SES circumstances (Howard et al. 2020, Hackman et al. 2015).

Importantly, the assumption that socioeconomic disadvantage uniformly constrains EF development may not hold consistently across cultural and ecological contexts. Much of the evidence supporting SES‐EF gradients derives from studies conducted within single‐country or high‐income Minority World contexts. This raises important questions about the extent to which these developmental patterns generalise globally, and whether children growing up in Majority World settings may demonstrate context‐specific strengths or adaptive developmental patterns not predicted by conventional SES models. A recent study (Howard et al. 2020) suggests the latter and challenges the assumption that children in highly disadvantaged environments inevitably have poorer cognitive outcomes. Specifically, in a study of South African and Australian preschoolers, the most disadvantaged South African children outperformed middle‐SES Australian preschoolers on two of three EFs and better than high‐SES Australian preschoolers on inhibition (Howard et al. 2020). These findings provided an important foundation for subsequent work examining whether conventional SES‐EF gradients operate differently in South African and other Majority World contexts. Subsequent research in low‐income South African settings has further shed some light on the nuanced environmental predictors of EF. For instance, Cook et al.’s longitudinal investigation of associations of risk and protective factors for EF in vulnerable preschool children in South Africa, has shown that early childhood care and education (ECCE) attendance and the diversity of caregivers are more predictive of EF growth over time than traditional SES indicators like household income or parental education (Cook et al. 2024).

Milosavljevic et al.’s cross‐country comparisons of EF skills and their environmental predictors among South African and Gambian preschool children have shown that children in both contexts perform within age‐expected EF ranges, with minimal links to standard SES markers, underscoring the complexity of EF development in resource‐constrained settings (Milosavljevic et al. 2024). Complementary work by Cook et al. investigated the contribution of pre‐academic skills to EF in South African preschoolers, and highlighted that EF remains a robust predictor of pre‐academic skills, further solidifying its importance for school readiness (Cook et al. 2023). Further to this, Draper et al.’s study investigating the ecological factors predicting school readiness in South Africa has shown that child‐level factors—particularly ECCE access and child age—are stronger drivers of school readiness outcomes than household or community‐level variables in these settings (Draper et al. 2024).

Notably, while adversity is often presumed to impair self‐regulation, Cook et al.’s study on South African preschool children's violence exposure and self‐regulation found no direct association between violence exposure and EF‐related regulation, although coercive parenting did show a modest positive link, pointing to the need for culturally contextualized interpretations of risk (Cook et al. 2022). These studies’ findings raise critical questions about the factors that may protect (e.g., resilience to stressors) or promote (e.g., meaningful roles within the family) EF development, despite adversity in Majority World countries like South Africa. The findings of Howard et al.’s study (Howard et al. 2020) require replication, and factors associated with EF advantages in disadvantaged contexts require further investigation.

Taken together, this body of work suggests that associations between SES and EF may operate through multiple interacting developmental pathways rather than through socioeconomic position alone. Distal structural factors (e.g. household resources, caregiver education, and community adversity), may shape children's EF indirectly through more proximal experiences within the child's immediate environment (e.g. caregiving practices, sleep quality, cognitive stimulation, daily responsibilities, and exposure to stress). Importantly, these pathways may not function uniformly across cultural and ecological contexts. In Majority World settings characterised by chronic adversity, some environmental demands may simultaneously constrain development while also fostering adaptive self‐regulatory capacities. Understanding how these interconnected factors operate within specific contexts is therefore critical for identifying both risk and resilience processes shaping early EF development.

While dominant developmental models posit a relatively universal SES gradient in EF development, these relationships remain insufficiently examined across diverse cultural contexts. South Africa offers a particularly important setting in which to investigate this, as emerging evidence suggests that children in low‐SES communities may exhibit EF strengths not typically observed among disadvantaged groups from Minority World countries. Such findings raise broader theoretical questions regarding resilience, adaptive calibration to adversity, and the extent to which conventional socioeconomic indicators adequately capture the developmental conditions shaping EF in Majority World settings.

This study therefore sought to both replicate and extend prior South African findings on EF development in low‐income settings. Specifically, we investigated how distal socioeconomic conditions may shape EF development through proximal developmental processes, such as parenting practices, sleep patterns, daily activities, and exposure to adverse events like violence, contribute to EF development in preschoolers from low‐income settings. By integrating these multiple contextual influences within a single framework, we aimed not only to identify contextually relevant predictors of EF, but also to contribute to broader developmental debates regarding resilience, adaptation, and the generalizability of SES‐based models of cognitive development.

Based on prior South African findings, we first sought to examine whether conventional socioeconomic indicators would show direct associations with preschoolers’ EF performance in this context. We further expected that more proximal developmental processes, particularly caregiver educational attainment and child sleep quality, would show stronger associations with EF outcomes. Given the emerging but mixed evidence regarding factors such as parenting practices, screen use, exposure to violence, and chronic stress in Majority World settings, analyses involving these variables were considered more exploratory and aimed to investigate potential context‐specific pathways associated with EF development under adversity. Exploratory analyses were also conducted on the subset of participants with hair cortisol concentration data to examine potential associations between chronic physiological stress and EF.

## 2 Methods

### 2.1 Study Design

This study employed a cross‐sectional design to investigate the predictors of EF development in South African preschool children from low‐income communities. A total of 125 caregiver–child dyads were recruited from preschools in Cape Town, South Africa, of which 120 children completed the full EF battery and were included in the main analyses.

### 2.2 Population and Sampling

Participants included caregiver‐child dyads who were recruited from a total of 15 schools from diverse low‐income communities within the Cape Town metropolitan area. The communities consist of both formal and informal housing. Informal dwellings are defined as makeshift structures, not approved by the local authority, and not meant for permanent residence. Overcrowding remains a significant issue with approximately 146,000 households residing in 437 informal settlement pockets across Cape Town (Isandla Institute 2024). As of the third quarter of 2024, the Western Cape's official unemployment rate stood at 19.6%, with an expanded unemployment rate of 25.6% (Isandla Institute, 2024). These communities face compounded challenges, including food insecurity, alcohol abuse, crime, and a high burden of HIV (Isandla Institute, 2024). Gang activity and drug‐related violence are also prevalent in certain areas (Isandla Institute, 2024).

Recruitment of children and primary caregivers (>18 years) was facilitated through a community‐based organisation that hosts workshops for principals and practitioners of early childhood development (ECD) centres. The research team presented the study during a workshop and invited the ECD centres to join the study. Information about the study was also put on a WhatsApp group for those who could not attend the workshop in person. ECD centres who were willing to participate distributed the information sheets and consent forms to caregivers of children aged 3–5 years in their respective schools.

### 2.3 Procedure and Measures

To address both the study objectives, data were collected separately from the sampled children and their caregivers. To address the first objective, namely, to replicate the finding of EF advantage in low‐SES South African contexts, we measured the sampled children's EF and early learning outcomes. To address the second objective, namely, to investigate the predictors of EF development in this context, caregivers were interviewed telephonically using surveys on home learning environment, parental education, socioeconomic indicators, daily child responsibilities, and exposure to community violence. Data collection was done by trained fieldworkers who could speak the home language of the participants (i.e., isiXhosa, English, or Afrikaans) and instructions for the EF tasks were translated into the appropriate home language.

First, we describe the child measures used to investigate child EF and early learning outcomes.

Executive Function. We used three iPad‐based EF measures from the Early Years Toolbox (EYT), a measurement instrument which has been translated into 8 South African languages, validated, and used extensively with preschoolers both in South Africa and globally. Research has also documented the appropriateness of this tablet platform for administering these measures, as it did not introduce biases associated with technological expertise or familiarity (Cook et al. 2023, Howard and Melhuish 2017). The EYT EF measures included:

1. Mr Ant (Working Memory). Children are presented with an image of a cartoon ant with several coloured dots placed in different spatial locations on its body. After a predetermined time limit these dots disappear and the child is asked to recall the spatial locations of the dots by tapping the corresponding locations on the ant's body. The number of dots increases and performance indices aim to estimate capacity of working memory. This task takes up to 8 minutes to complete.1.
2. Go/No‐Go (Inhibition). Children are presented with fish and sharks swimming across the screen and instructed to tap the screen when they see a fish (‘catch the fish’) and refrain from tapping when a shark appears (‘avoid the sharks’). Indices reflect the product of no‐go and go accuracy, such that higher scores reflect stronger impulse control. This task takes 5 minutes to complete.2.
3. Card Sort (Shifting). Children are presented with ‘cards’ that vary along two dimensions (e.g., shape and colour) and are asked to sort each card (e.g., red rabbits and blue boats) first by one dimension (e.g., colour) and then, after some trials, by another dimension (e.g., shape). Indices reflect the correct number of sorts after the first shift in rules, with higher scores indicating better shifting performance. This task takes up to 6 minutes to complete.3.

In line with recent recommendations (Camerota et al. 2020), a composite variable approach was used to generate the total EF score by standardising the scores from each task and averaging them into a single composite score. Inter‐task correlations, exploratory factor analysis, and composite reliability estimates (omega total, ω_t_) were conducted subsequently as validation procedures to evaluate whether the three EF tasks demonstrated sufficient coherence to support use of a composite EF measure, as reported in the Results. Cronbach's α was not used, as EF tasks are not parallel items of a reflective construct.

Child assessments were administered by local fieldworkers who were familiar with the context, spoke the local language, and had been trained in administering these measures. Children completed the assessments in a relatively quiet area of their preschool that was open to visual and auditory supervision by centre staff. Assessments were completed in a single 15‐20‐minute session.

Demographic Data. Seventeen questions to ascertain: the child's age, gender, birth weight, pattern of preschool attendance, number of adults and children in the household, primary caregiver's age, marital status, place of birth, education, primary home language, additional languages spoken within the household (i.e., multilingual exposure), and household employment and income (selected items from the National Income Dynamics Survey, Wave 1).

Child's Screen Time. A 9‐item questionnaire on the child's screen behaviours (Isandla Institute 2024), including the amount of time using screen devices, the frequency of use for different purposes, and proximity of use to the child's bedtime. We calculated the total screen time score and screen regulatory use (α = 0.93).

Child's Sleep. A 27‐item questionnaire on the child's typical bedtime and wake time, sleep time, and sleep‐time behaviours/routines (e.g., whether the child moves to someone else's bed during the nighttime) (Owens et al. 2000). We calculated a sleep difficulties score based on items showing problems with bedtime, wake time and sleep time (α = 0.83).

Child's Daily Activities and Responsibilities. This information was collected via an adapted time‐use diary, reported for the last weekday and last weekend day. Parents had to report child activities within each time interval, with prompts regarding possible types of activities and responsibilities. Examples included helping with household chores (e.g., tidying, assisting with younger siblings), participation in family routines, independent self‐care activities, play, and educational activities. Activities were categorised to determine the frequency, duration and patterns of each child's daily activities. This measure was intended to capture children's engagement in everyday household and family responsibilities, which in some contexts may reflect adaptive autonomy and participation in family life, but may also reflect increased expectations or burden under conditions of socioeconomic adversity. This activity was adapted from scales that are used routinely in global large‐scale longitudinal studies (Corey et al. 2014). Although our previous research indicated the potential for these daily activities and responsibilities to have predictive value for EF, given the performance of the measure, we opted only to provide descriptive data on these variables.

Child's Exposure to Violence. The 29‐item Child Exposure to Community Violence (CECV) scale measures whether and/or the extent to which a child has witnessed general violent or criminal acts, experienced family and non‐family violence, and feelings or experiences of unsafety (Cook et al. 2022, Martin et al. 2012, Donald et al. 2019). The 29‐item CECV has been used in other South Africa studies of preschool‐age children from low‐income settings (Cook et al. 2022, Donald et al. 2018). Questions are answered on a scale of never, once, twice, three to ten times, or more than ten times. Scores are summed to yield a total score, with a higher total score indicating a higher volume of exposure to community violence. As the items in the CECV are not parallel indicators of a single latent construct, internal consistency statistics such as Cronbach's α are not appropriate. These items form a formative rather than reflective construct, where each type of exposure contributes uniquely to the cumulative violence experience rather than representing interchangeable indicators of a single latent factor. While composite reliability (e.g., omega total, ω_t_), is appropriate for reflective constructs such as EF tasks, it is not meaningful for formative indices like cumulative exposure. Consistent with prior applications of the CECV in South African preschool samples, we therefore used summed scores to index total exposure.

Parenting and Parent Mental Well‐Being. The 30‐item Parenting and Family Adjustment Scale (PAFAS) investigates parent interactions with the child (e.g., I chat/talk with my children), parenting behaviours (e.g., If my child doesn't do what they're told to do, I give in a do it myself) and parental sentiments (e.g., I am proud of my child). It includes some questions that relate to better or worse mental well‐being (e.g., I feel stressed or worried; I feel satisfied with my life) (Donald et al. 2019, Sanders et al. 2014). Scores are summed to yield a total score, as well as subscale scores for: parental consistency, coercive parenting, positive encouragement, caregiver‐child relationship, family adjustment, and parental adjustment. For each subscale, items were summed such that a higher score indicates higher levels of dysfunction. For PAFAS, internal consistency for the total PAFAS score was α = 0.79, showing good overall reliability. At the subscale level, the caregiver–child relationship (α = 0.76) and family relationship (α = 0.75) demonstrated acceptable internal consistency and were therefore retained for analyses. Other subscales showed inadequate reliability (α = 0.54–0.69) and were excluded from further analyses.

Chronic Stress. As an additional optional study component, hair samples were collected directly from consented participating children to derive a hair cortisol concentration index as an indicator of chronic stress. Where parents consented to this component, a hair sample of at least 2 cm of hair was collected for cortisol level analysis. Hair samples were prepared and analysed as described by Meyer et al.2014 with minor modification. Briefly, hair samples were washed twice in isopropanol and allowed to dry for 2 days to ensure complete evaporation of remaining isopropanol. A 3 cm long section of each hair sample (taken proximally from the scalp), weighing between 20 and 65 mg, was finely diced into a powder using a surgical scalpel blade. The powdered hair was extracted in 1 mL of methanol for 24 hours at room temperature, with gentle shaking. Samples were centrifuged at 10,000 rpm for 5 min at room temperature to pellet the powdered hair, and 600 µl of supernatant was removed to a clean tube. Following evaporation of methanol, extracts were reconstituted in 250 µl assay buffer from a commercial cortisol ELISA kit (Arbor Assays, MI, USA, Catalog K003‐H1). Samples were analysed using the commercial ELISA kit, in duplicate, according to the manufacturer's protocol. All standards, controls, and samples were assayed in duplicate, and results were calculated using a 4‐parameter logistics curve fit. To convert assay values (ug/dl) to pg/mg of hair, the following equation was used:

$$\left(A/B\right)*\left(C/D\right)*E*10,000=F$$

where A = µg/dl from assay output; B = weight (in mg) of hair used; C = vol. (in ml) of methanol added to each sample; D = vol. (in ml) of methanol recovered and dried; E = vol. (in ml) of assay buffer used to reconstitute extract; and F = final value of hair cortisol concentration in pg/mg.

This analysis allowed the derivation of an index of the cortisol (i.e., chronic stress) levels of the child. These samples were anonymised (using child ID numbers) and sent for cortisol analysis at the University of New England's lab (a Co‐I on this study) in Australia.

### 2.4 Data Analysis

All analyses were conducted using Stata 18. Descriptive statistics were computed to summarize sample characteristics and key study variables. Continuous variables were reported as means and standard deviations (M ± SD), while categorical variables were summarized as frequencies and percentages.

We first conducted correlation analyses to broadly explore associations between variables. Continuous variables were screened for distributional assumptions prior to analysis. Hair cortisol concentration exhibited substantial positive skewness and was therefore analysed using Spearman's rank‐order correlation, whereas the remaining variables were judged to meet assumptions sufficiently for Pearson correlations and linear regression. After this, to examine the associations between executive function (EF) outcomes and relevant predictors, multiple linear regression analyses were performed. Final models were evaluated based on statistical significance (p < 0.05) and adjusted R^2^. All regression models were tested for multicollinearity using the Variance Inflation Factor (VIF), ensuring that predictor correlations did not inflate standard errors. Robust standard errors were used to account for potential heteroskedasticity. All statistical tests were two‐tailed, with significance set at *p* < 0.05.

Sample sizes varied across measures because not all participants had complete data for every assessment or caregiver‐reported measure. Of the 125 caregiver–child dyads recruited, 120 children completed the full EF battery and constituted the main EF analytic sample. The reasons for individual instances of missing data were not systematically recorded in the dataset available to the authors and therefore could not be reliably classified retrospectively (e.g., as absence, withdrawal, or non‐response). Regression analyses were conducted using complete cases, such that participants with missing data on any variable included in a given regression model were excluded from that model. Both the full and reduced regression models were therefore based on the same analytic sample of 103 participants. Thus, 17 participants with complete EF data were excluded from the regression analyses because of missing data on one or more predictor variables.

### 2.5 Ethical Considerations

The study was approved by the Human Research Ethics Committee (Medical) at the University of the Witwatersrand (reference number is M190962). This study adheres to the guidelines described in the Declaration of Helsinki Ethical Principles for Medical Research Involving Human Subjects, the International Conference on Harmonisation and the European Good Clinical Practice (GCP) guidelines, the South African GCP guidelines, and the laws of South Africa.

## 3 Results

### 3.1 Descriptive Statistics

Tables 1 and 2 report descriptive statistics for all analytic variables. In terms of measurement reliability, EF measures (i.e. EYT) showed moderate positive inter‐task correlations in line with prior findings—ranging from r = 0.13 to 0.33 (N = 116)—which supported a composite approach to their combination. An exploratory factor analysis showed that a single factor accounted for 51% of the variance across tasks. Standardized factor loadings were moderate to strong (inhibition λ = 0.66, cognitive flexibility λ = 0.66, working memory λ = 0.81). Composite reliability (ω_t_ = 0.76) showed adequate reliability of the EF composite score.

**TABLE 1:** Descriptive statistics for continuous analytic variables.

	Mean ± SD	Median	Range
*Demographics*			
Child age (*N* = 125), 49.6% girls	4.64 ± 0.79	4.69	3.13–5.79
Household asset score (*N* = 125)	11.13 ± 2.63	11.00	5–18
*Child sleep*			
Total hours of sleep (*N* = 121)	10.74 ± 1.67	11.00	2.5–15
Sleep difficulties score (*N* = 125, *α* = 0.83)	1.31 ± 0.36	1.81	1–3
*Child screen time*			
Total hours of screen time (*N* = 110)	2.62 ± 1.36	2.00	0.5–6
Screen regulatory use (*N* = 125, *α* = 0.93)	1.53 ± 1.45	1.20	0–4
*Child exposure to community violence*			
Total CECV score (*N* = 121)	2.77 ± 3.78	1.00	0–17
*PAFAS*			
Caregiver‐child relationship (*N* = 125, *α* = 0.76)	0.96 ± 1.88	0.00	0–15
Family relationship (*N* = 125*, α* = 0.75)	1.70 ± 2.11	1.00	0–11
*Daily activities on a week and a weekend day*			
Enrichment activities (*N* = 130)	1.26 ± 1.32	1.00	0–6
Screentime (*N* = 130)	2.28 ± 1.12	2.00	0–5
Play (*N* = 132)	1.15 ± 1.05	1.00	0–4
Socialising (*N* = 131)	0.87 ± 0.89	1.00	0–3
Responsibilities and routines (*N* = 131)	0.93 ± 1.06	1.00	0–5
*Executive function*			
EF composite score (*N* = 116, *ω_t_* = 0.76)	−0.04 ± 0.70	−0.06	−1.60–1.88
Hair cortisol (*N* = 25)			
Ug/dl direct from assay	1101.60 ± 853.78	1023.61	242.06–3824.11
Conversion to pg/mg hair	14.40 ± 9.68	12.39	4.05–37.55

*Note*: PAFAS—Parental and family adjustment scales. CECV—Child exposure to community violence. Sample sizes vary across variables due to missing responses.

**TABLE 2:** Descriptive statistics for categorical analytic variables.

	Count (% of sample)
*Demographics*	
Child sex (*N* = 125)	Male: 63 (50.40%)
	Female: 62 (49.60%)
*Caregiver education*	
Matric certificate (*N* = 124)	Yes: 97 (78.23%)
	No: 27 (21.77%)
Post‐school qualification (*N* = 120)	None: 91 (75.83%)
	Certificate: 12 (10.00%)
	Diploma: 10 (8.33%)
	Degree: 7 (5.83%)

*Note*: Sample sizes vary across variables due to occasional missing caregiver responses. Percentages are calculated based on available data for each variable.

### 3.2 Preliminary Exploratory Correlation Analyses

Correlation analyses were conducted as a preliminary, exploratory assessment of associations among the study variables and to inform the subsequent regression analyses. Table 3 displays the correlation matrix of all measured variables. These correlations were intended to provide a descriptive overview of the relationships among variables rather than to constitute a series of standalone confirmatory hypothesis tests.

**TABLE 3:** Correlations among study variables.

Variable	1	2	3	4	5	6	7	8	9	10	11	12	13	14
1. Child age	—													
2. Parent secondary education	−0.05	—												
3. Parent tertiary education	0.14	0.20	—											
4. People in household	0.23	0.12	−0.14	—										
5. Screen time	0.08	0.08	−0.01	0.12	—									
6. Screen regulatory use	0.07	0.08	−0.02	0.03	−0.50	—								
7. Total hours of sleep	−0.09	0.00	0.00	0.02	0.27	−0.60	—							
8. Sleep difficulties	0.10	0.07	0.00	0.05	0.32	0.36	0.33	—						
9. Stress total score (CECV)	0.06	−0.07	−0.10	−0.02	−0.15	−0.13	−0.06	0.35	—					
10. Caregiver‐child relationship	0.03	0.00	−0.19	−0.15	−0.06	0.01	−0.07	0.08	0.28	—				
11. Family relationship	0.08	0.08	−0.22	0.13	−0.18	0.23	−0.21	0.45	0.28	−0.05	—			
12. Parent scales total score (PAFAS)	0.08	−0.02	−0.25	−0.16	−0.28	0.54	−0.46	0.51	0.33	0.47	0.50	—		
13. Household items score	0.01	0.29	0.26	0.20	−0.28	0.64	−0.38	0.29	−0.23	0.00	0.05	0.29	—	
14. EF composite score	0.38	0.25	0.17	−0.02	0.08	−0.02	−0.02	−0.17	−0.09	0.03	−0.14	−0.07	0.05	—

Note: CECV = Child Exposure to Community Violence; PAFAS = Parenting and Family Adjustment Scales. Values represent Pearson correlation coefficients. Only the lower triangle of the correlation matrix is presented to avoid redundancy.

As shown in Table 3, child age showed a positive association with EF (*r* = 0.38, *p* < 0.001), indicating that older children performed better on EF tasks. Caregiver secondary education was positively associated with EF (*r* = 0.25, *p* = 0.005).

### 3.3 Multiple Linear Regression Analysis

Multiple linear regression analyses were used to examine which theoretically relevant factors predicted children's EF scores. Variance inflation factors indicated no issues of multicollinearity (all VIF < 2). The individual predictors with their beta values are shown in Tables 4 (full model) and 5 (after stepwise model reduction). Although 120 children completed the full EF battery, the regression analyses were based on 103 complete cases because of missing data across the predictor measures. The full and reduced regression models were both estimated using this same analytic sample (*N* = 103).

**TABLE 4:** Summary details of the multiple linear regression analyses (full model) for of variables predicting performance on EF outcomes.

Predictor	*Β*	SE β	95% CI	*t*	*p*
Child age	0.37	0.09	[0.21, 0.54]	4.40	<0.001^a^
Caregiver secondary education	0.22	0.07	[0.07, 0.36]	3.02	0.003^a^
Caregiver tertiary education	0.10	0.17	[–0.25, 0.44]	0.55	0.581
Screen time	−0.02	0.05	[–0.12, 0.08]	−0.41	0.680
Sleep difficulties	−0.45	0.22	[–0.88, –0.02]	−2.06	0.042^a^
Stress exposure total score (CECV total score)	−0.00	0.02	[–0.04, 0.04]	−0.05	0.957
Family relationship score	−0.01	0.04	[–0.09, 0.06]	−0.37	0.709
Parent scales total score (PAFAS score)	0.01	0.01	[–0.02, 0.03]	0.42	0.676
Household items score	0.00	0.03	[–0.06, 0.06]	0.02	0.987
Model fit: *F*(9, 93) = 3.95, *p* < 0.001; *R* ^2^ = 0.28; Adj. *R* ^2^ = 0.21.					

^a^ Statistically significant predictors at *p* = 0.05.

**TABLE 5:** Summary details of the multiple linear regression analyses (stepwise model) for of variables predicting performance on EF outcomes.

Predictor	*β*	SE *β*	95% CI	*t*	*p*
Child age	0.38	0.08	[0.23, 0.54]	4.81	<0.001
Caregiver secondary education	0.22	0.06	[0.09, 0.35]	3.43	0.001
Sleep difficulties	−0.42	0.16	[–0.74, –0.09]	−2.55	0.012
Model fit: *F*(3, 99) = 12.18, *p* < 0.001; *R* ^2^ = 0.27; Adj. *R* ^2^ = 0.25.					

The full model (Table 4) including nine predictors was significant, *F*(9, 93) = 3.95, *p* < 0.001, explaining 21% of the variance in EF (*R* ^2^ = 0.21). Child age (*β* = 0.37, *p* < 0.001), caregiver secondary education (*β* = 0.22, *p* = 0.003), and child sleep difficulties (*β* = –0.45, *p* = 0.042) emerged as significant predictors. Caregiver tertiary education, screen time, exposure to community violence (stress total score), family relationship, parenting and family adjustment total score (total PAFAS score), and household assets were non‐significant (all *p* > 0.05).

A stepwise process of model reduction (Table 5) confirmed the best‐fitting model retained three predictors, namely child age (*β* = 0.38, *p* < 0.001), caregiver secondary education (*β* = 0.22, *p* = 0.001), and sleep difficulties (*β* = −0.42, *p* = 0.012), which together explained 27% of the variance in EF (*R* ^2^ = 0.27; adjusted *R* ^2^ = 0.25), *F*(3, 99) = 12.18, *p* < 0.001.

### 3.4 Exploratory Analysis of Hair Cortisol Data

To assess potential selection bias in the hair cortisol subsample, participants who provided hair samples were compared with those who did not, based on key demographic, socioeconomic, adversity, educational, and EF variables. No significant differences were observed for child age (*p* = 0.420), household asset score (*p* = 0.164), exposure to community violence (*p* = 0.992), caregiver educational attainment (school education: *p* = 0.760; post‐school education: *p* = 0.472), or EF performance (*p* = 0.369). However, children providing hair samples reported significantly greater child sleep difficulties than those who did not provide samples (*p* = 0.011). Overall, these findings suggest that the hair cortisol subsample was broadly representative of the larger study sample, although some participation bias related to sleep difficulties cannot be ruled out.

Hair cortisol concentration (HCC) data were available for a subset of 25 participants. However, when investigating the relationship between HCC and EF, only 22 cases had both HCC and EF data (*n* = 22). Hair cortisol concentrations ranged from 4.05 to 37.55 pg/mg hair (*M* = 14.40, SD = 9.68; median = 12.39), indicating substantial variability in chronic physiological stress within the sample. Given the absence of universally accepted clinical cut‐offs for preschool‐aged children and methodological differences across studies, HCC values are best interpreted as indicators of relative variation in chronic physiological stress rather than as markers of clinically high or low cortisol levels.

Hair cortisol concentration was positively associated with EF scores. Pearson's correlation indicated a medium‐sized positive association (*r* = 0.42, *p* = 0.054, *n* = 22), which approached significance. Given the skewed distribution of HCC, we also examined Spearman's rank‐order correlation, which confirmed a significant positive association (*ρ* = 0.55, *p* = 0.009). These findings suggest a moderate association, such that higher HCC was related to better EF performance (and vice versa) in this subsample. However, these findings should be interpreted with caution due to the limited sample size and potential for bias in the consented sample (Figure 1).

**FIGURE 1:**
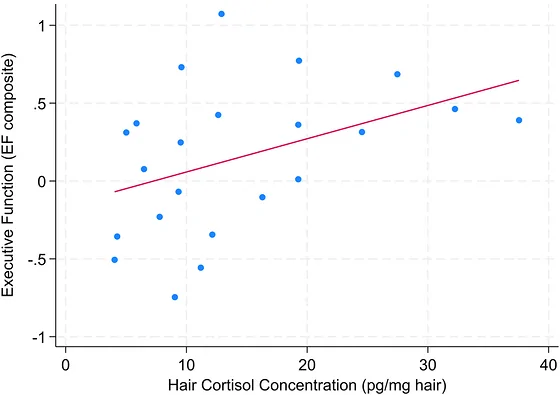
Hair cortisol conversion versus EF factor with Regression Line.

## 4 Discussion and Conclusions

This study demonstrated that preschool children from highly disadvantaged, low‐income South African communities had a complex pattern of EF predictors, which did not perfectly conform to patterns found in even low‐income Majority World contexts. Specifically, predictors of EF in this sample were child age, caregiver secondary education, and child sleep difficulties, whereas predictors identified in other contexts such as household wealth (Raffington and Prindle 2018, Kim et al. 2021, Barbee et al. 2025), did not significantly predict South African preschoolers’ EF. Our analyses revealed a complex pattern of associations, identifying robust predictors, potential indirect influences, and several factors that, contrary to literature from high‐income countries, showed no significant relationship with EF in this context. Importantly, these findings should not be interpreted as indicating that the identified predictors operate independently of one another. Consistent with ecological and developmental systems perspectives, factors such as caregiver education, household resources, parenting practices, sleep quality, and exposure to adversity are likely interconnected and may influence EF through multiple indirect and reciprocal pathways. The present analyses were designed to identify factors most strongly associated with EF within this sample rather than to formally test developmental mechanisms or mediational processes. Future longitudinal studies using larger samples will be important for examining how distal socioeconomic conditions shape EF through more proximal developmental experiences.

a. Significant Predictors of EF

Across both correlation and regression analyses, three factors, namely child's age, caregiver secondary education level, and child sleep difficulties were strong predictors of child EF. The strong positive association with age (r = 0.38; β = 0.37, p < 0.001) aligns with well‐established developmental trajectories, confirming that core neurocognitive maturation proceeds even in the presence of socioeconomic adversity (Xu et al. 2023, Bick et al. 2018, Tsunga et al. 2024, Draper et al. 2025). More notably, caregiver secondary education emerged as a significant positive predictor (*r* = 0.25; *β* = 0.22, *p* = 0.003), though weaker than might be expected in higher‐income settings (Vrantsidis et al. 2020, Xu et al. 2023, Bick et al. 2018), while household asset score (HAS), a traditional marker of SES showed no such relationship (*r* = 0.03). The absence of a tertiary education effect may partly reflect limited variability within this sample, as only 24% of caregivers reported any tertiary level qualification. In highly disadvantaged communities, completion of secondary education may therefore represent a meaningful developmental threshold, distinguishing caregivers who have acquired literacy, problem‐solving skills, and structured learning experiences that support cognitively enriching parenting. By disaggregating secondary and tertiary education, rather than using a single composite index, we were able to explore whether incremental educational levels confer distinct benefits in this context. The findings suggest that while secondary education was meaningfully associated with children's EF, additional formal education may not translate into further advantages under persistent socioeconomic constraint. This distinction suggests that it might not be material wealth, but rather the knowledge, skills, and practices associated with higher caregiver secondary level education that foster EF development. Education empowers caregivers to create a more cognitively stimulating home learning environment and engage in parenting practices that supports EF development, echoing findings from other studies in similar contexts (Rubio‐Codina et al. 2016, Son and Morrison 2010, Cuevas et al. 2014, Cook et al. 2023).

Furthermore, sleep difficulties demonstrated a significant, small‐to‐moderate negative association with EF (*β* = −0.45, *p* = 0.042). This trend reinforces the growing body of evidence linking adequate sleep to better cognitive and self‐regulatory outcomes in early childhood ((Drummond et al. 2012, Chaput et al. 2017), 81, 82), positioning it as a key modifiable factor for intervention.

b. Factors Not Significant in Either Analysis: Challenging Assumptions from High‐Income Contexts

Additional nuance was found in theoretically relevant and empirically supported (within other contexts) factors that showed no significant association with child EF, contrasting the balance of evidence from high‐income, Minority World contexts. First, the lack of a clear gradient between household asset score and EF directly (*r* = 0.03) challenges the presumed universality of this relationship and replicates prior work in South Africa (Howard et al. 2020, Cook et al. 2023). It suggests that in highly disadvantaged but resilient communities, proximal processes (e.g., what parents do with their resources) outweigh the influence of material wealth alone. Importantly, these findings should not be interpreted as evidence that socioeconomic conditions are irrelevant for EF development. Rather, household asset ownership did not significantly predict EF within this relatively socioeconomically constrained sample. Socioeconomic disadvantage may influence EF through multiple interconnected pathways, including caregiver education, parenting practices, sleep quality, exposure to adversity, access to educational opportunities, and the quality of children's daily developmental experiences. The significant associations observed for caregiver education and sleep difficulties may therefore represent more proximal mechanisms through which broader socioeconomic conditions shape EF development. Future research should also examine other potentially influential but unmeasured proximal processes, such as the degree of children's household chores and responsibilities and parental expectations for compliance, which may shape EF development in ways not captured by standard socioeconomic indicators.

Second, we found a weak non‐significant association between exposure to community violence and EF (*r* = −0.09). Similar findings have been found by Cook et al. (Cook et al. 2022) and Tsunga et al. (2024). In this sample, more than half of the children (70/121, 57.9%) reported exposure to at least one community violence event. This prevalence indicates that violence exposure was common within the study population and suggests that the null association is unlikely to stem from uniformly low exposure or underreporting. Rather, it may point to context‐specific resilience or adaptation to adversity. Repeated exposure to unpredictable or threatening environments can require heightened vigilance, impulse control, and flexible problem‐solving (capacities that overlap with core EF processes). Children in such contexts may thus develop EF strengths that enable effective functioning under chronic stress. This interpretation aligns with recent work suggesting that adversity does not uniformly impair self‐regulation, but may sometimes shape EF development in adaptive ways depending on the environmental demands and socialisation patterns of the community (Draper et al. 2025). It underscores the critical need for culturally contextualized interpretations of risk, as not all adversities impact development in the same way across cultures (Cook et al. 2022).

c. Exploratory Evidence from Hair Cortisol Analysis

Although based on a small subsample (*n* = 22 with both EF and HCC data), exploratory analyses of children's HCCs, a biomarker of chronic stress exposure, showed that a moderate positive association between HCC and EF scores (*r* = 0.42, *p* = 0.054; Spearman's *ρ* = 0.55, *p* = 0.009). These results provide further support for the above provocation that EF‐promoting conditions might be found in disadvantaged contexts. Within this South African sample, higher chronic physiological stress was linked to comparatively better EF performance. This counterintuitive pattern may indicate adaptive calibration to challenging environments, where children exposed to persistent stress develop enhanced capacities to navigate demanding contexts. However, given the limited sample size and the exploratory nature of these analyses, these results should be interpreted cautiously.

Comparison of children who provided hair samples with those who did not, showed that the subsample was broadly comparable to the wider study sample across demographic, socioeconomic, adversity, educational, and EF measures. However, children whose caregivers consented to hair sampling showed slightly more child sleep difficulties, suggesting the possibility of limited participation bias. The exploratory cortisol findings should therefore be interpreted with appropriate caution and replicated in larger samples.

### 4.1 Implications

Our findings replicate and extend previous work by Howard et al. (2020) and Cook et al. (2023), demonstrating that SES does not directly predict EF outcomes among South African preschoolers. Rather than suggesting that SES is unimportant, our results suggest that EF development in South African preschoolers may be shaped more by proximal and potentially intervention‐sensitive factors (i.e., caregiver educational attainment, sleep, caregiving) than by household wealth or exposure to adversity. While caregiver educational attainment is not easily modifiable within established family systems, it may index a grouping of alterable caregiving and home‐environment processes that are more directly amenable to intervention. Indeed, there was at least suggestive evidence adversity—or more likely some factors associated with adversity (e.g., high and meaningful responsibilities given to children, children's need for vigilance, high expectations for compliance)—might have some positive association with EF. In this way, our findings challenge some of the assumptions derived from Minority World contexts and provides evidence that context‐specific pathways of resilience and adaptation are central to understanding child development in Majority World settings.

From a research perspective, our study reinforces the necessity of moving beyond broad SES correlates to investigate specific, modifiable mechanisms. It also highlights the need for culturally adapted and reliable measurement tools, as the low internal consistency of some measures (e.g., violence exposure) limits interpretability. Unlike the EF composite, which reflects a cohesive latent construct and thus benefits from reliability indices such as ω_t_, the violence exposure measure captures diverse but non‐interchangeable experiences. Its formative nature limits the usefulness of internal consistency metrics, underscoring the need for alternative reliability frameworks tailored to contextually complex constructs. From the perspective of practice and policy, our findings suggest that policymakers should prioritise expanding access to quality ECCE and supporting parental education initiatives. Interventions do well to include efforts and information that engender supportive caregiver‐child interactions and healthy sleep routines, as these modifiable factors showed the strongest evidence for supporting EF development in these low‐income settings.

### 4.2 Limitations and Future Directions

Regarding the limitations of our study, the cross‐sectional design precludes causal inferences. Generalisability of our findings may be restricted to similar peri‐urban, low‐income South African populations. In addition, the relatively restricted socioeconomic range of the sample may have limited our ability to detect broader socioeconomic gradients in EF. Because participants were recruited from predominantly low‐income communities, there may have been insufficient variability in household socioeconomic circumstances to observe effects that have been reported in more socioeconomically diverse samples. Yet, at the same time, novel and nuanced insights from under‐researched contexts is important to confirm, challenge, or nuance EF theorising. An additional limitation is that the exploratory correlation matrix involved multiple pairwise comparisons, and no correction for multiple testing was applied. The correlations were intended as a preliminary, descriptive assessment to inform the subsequent regression analyses rather than as a series of independent confirmatory tests. The key associations identified in the correlation analysis were subsequently examined in the regression models, providing additional support for their relevance within this sample. Our findings also highlights the need for culturally adapted and contextually validated measurement tools, as demonstrated by initiatives such as the LEVANTE project (Jukes et al. 2024). Such work underscores the importance of developing psychometrically sound instruments that reflect the lived realities of Majority World settings. Nevertheless, future research would benefit from longitudinal, multi‐site designs with larger samples and more robust contextual measurements.

## 5 Conclusions

Preschoolers from disadvantaged South African communities had EF skills that were associated most strongly with modifiable proximal factors (particularly child age, parental education, and sleep), rather than by SES per se. This pattern of results challenges the universality of the SES‐EF gradient and underscores that development, even under adversity, is promoted by specific, alterable processes within the child's immediate environment. Yet some influential factors that have been identified in previous research in other contexts were not associated with EF in this context, suggesting that our understandings of what influences children's EF growth, for whom, when, and under what conditions still needs to be better understood. Although previous studies have shown that supporting ECCE attendance and valuing parental education should be central to early development interventions, our study further highlighted the importance of factors related to caregiver‐child relationships and encouraging healthy routines including sleep habits.

## Author Contributions

All authors developed the study idea, designed the research methodology, and collected the data. C.D. and J.B. developed the initial conceptualisation of the manuscript. J.B. prepared the drafts of the manuscript and conducted the analysis. All authors contributed to the editing of the manuscript, as well as approval of the final manuscript for submission.

## Funding

This study was supported by competitive internal grant funding from the University of Wollongong. J.B. is supported by a postdoctoral fellowship from the Department of Science and Innovation and the National Research Foundation Centre of Excellence in Human Development at the Witwatersrand, Johannesburg, South Africa.

## Ethics Statement

The study was approved by the Human Research Ethics Committee (Medical) at the University of the Witwatersrand (reference number is M190962).

## Conflicts of Interest

There is no conflict of interest in this project.

## Data Availability

Data supporting the findings of this study are available upon reasonable request from the corresponding author. The data are not publicly available due to ethical restrictions related to participant confidentiality.
